# A quarter pound of acetaminophen with propylene glycol on the side: A case report 

**DOI:** 10.5414/CNCS109936

**Published:** 2020-09-01

**Authors:** Daniel Murphy, Abrar Khan, Christine Borscheid, Samy Riad

**Affiliations:** 1 Department of Medicine and; 2 M Health Fairview, University of Minnesota, Minneapolis, MN, USA

**Keywords:** acetaminophen, overdose, hemodialysis

## Abstract

Particularly large acetaminophen overdoses, termed massive, create a therapeutic challenge given the standardized, N-acetylcysteine-based treatment. One consideration in addition to N-acetylcysteine is the initiation of hemodialysis due to the dialyzable nature of acetaminophen, though encumbered by the concurrent removal of the antidote, N-acetylcysteine. Such cases of large acetaminophen overdose, along with possible concomitant ingestions of other drugs or inactive ingredients, can be complicated by challenging-to-interpret clinical signs and laboratory findings. We describe a case of a 46-year-old man for whom we were consulted regarding consideration of dialysis treatment 7 hours after ingestion of 125 g of acetaminophen. The patient developed multiple early signs and laboratory findings consistent with a significant acetaminophen overdose. He also developed a rarely described, likely acetaminophen-interference-induced laboratory abnormality. Finally, he possibly had toxicity from an “inactive” ingredient. He was treated with a single session of prolonged hemodialysis (9.5 hours) and increased dosing of N-acetylcysteine with a positive outcome. Herein, we discuss the decision making and interpretation of clinical data pertaining to dialysis treatment and other therapies after a massive acetaminophen overdose.

## Introduction 

Acetaminophen toxicity is a common cause of acute liver injury and failure [1]. In cases of large ingestions, early metabolic and renal derangements, including severe lactic acidosis, may also occur due to systemic glutathione depletion and mitochondrial dysfunction [2, 3]. Cases of massive acetaminophen ingestion, exceeding 0.5 g/kg, have been treated with hemodialysis in addition to N-acetylcysteine due to the dialyzable nature of acetaminophen [4]. Acetaminophen has a molecular weight of 151.2 Da, a volume of distribution of 0.8 – 1.0 L/kg, and is ~ 25% protein-bound. Dialysis can be done rather than solely treating with protocolized N-acetylcysteine, the dose of which is typically determined by the patient’s weight but not acetaminophen dose. Increased dosing of N-acetylcysteine must be considered in cases of markedly high acetaminophen doses of plasma levels due to increased risk of hepatotoxicity [5, 6, 7]. Furthermore, the dialyzable nature of N-acetylcysteine when used simultaneously with dialysis must also be considered. Should the patient have overt liver failure, low-intensity dialysis modalities would reduce risk for cerebral edema [8], though high-intensity dialysis would achieve greater toxin removal [4]. 

Pertinent to cases of acetaminophen overdose and diagnosing liver failure, lab abnormalities not reflective of acetaminophen’s hepatotoxic effects have been reported, including direct effects on the coagulation cascade causing early, modest INR elevations [9]. Similar coagulation effects have been reported for N-acetylcysteine [10, 11]. 

N-acetylcysteine has also been described as causing interference with creatinine measurement by the older Jaffe method [12]. While enzymatic methodologies of creatinine measurement are reported to be less likely to experience interference, multiple interfering substances have been described [13]. Our institution uses enzymatically measured creatinine by Siemens Dimension Vista System Flex reagent cartridges (Siemens Healthcare Diagnostics, Inc., Malvern, PA, USA). Interferences by either acetaminophen or N-acetylcysteine are described in the package insert as “minimal to none” at concentrations tested. While dialysis may be used to treat acetaminophen overdose regardless of renal function, acetaminophen can cause acute kidney injury, and assessment of renal function is still germane [3]. 

Finally, co-ingestions are often suspected in cases of acetaminophen overdose [14]. However, it is important to also consider additional ingredients in an overdosed medication. Toxicity and adverse reactions have been reported for “inactive” ingredients in multiple medications [15, 16]. Of note, some acetaminophen-containing products also include propylene glycol as an inactive ingredient [17]. 

## Case 

We consulted on a 46-year-old man weighing 84 kg 7 hours after ingestion of 125 g (two hundred fifty 500-mg pills) of acetaminophen with “inactive” propylene glycol as an ingredient and an unknown quantity of dextromethorphan. He presented within 1 hour of ingestion with resulting severe lactic acidosis and obtundation, for which he was intubated. Hours later, the patient developed hypotension and osmotic diuresis (urine osmolality of 532 mOsm/kg) with polyuria of 8 L in the first 24 hours with albuminuria and glucosuria. INR, 1.0 on presentation, had risen to 1.5 at time of consultation. Alanine transaminase (ALT) was 72 U/L (reference range: 0 – 45 U/L) with aspartate transaminase (AST) (reference range: 0 – 70 U/L), bilirubin, alkaline phosphatase, and serum ammonia levels within normal limits at that time. He had received “stage 1” and “stage 2” of protocolized N-acetylcysteine (150 mg/kg followed by 50 mg/kg) at an outside hospital before transfer to our institution. At presentation, serum creatinine was measured as 0.9 mg/dL, consistent with prior baseline creatinine. 

We prescribed hemodialysis with maximal blood flow and 600 mL/min dialysate flow rate for maximum clearance due to the massive ingestion without apparent, significant liver injury and an alternative explanation for his coagulopathy. INR remained stable for the duration of hemodialysis, and AST and ALT would peak at 88 U/L and 105 U/L, respectively. Bilirubin and alkaline phosphatase remained within normal limits throughout the case. 

Prior to initiation of either hemodialysis or “step 3” of protocolized N-acetylcysteine, the patient’s serum creatinine was measured twice at “< 0.14 mg/dL” and remained so during the duration of hemodialysis. In the setting of unknown glomerular filtration rate, the patient had both metabolic anion gap lactic acidosis and non-gap acidosis. Albuminuria was seen. Glucosuria was also seen with concurrent hyperglycemia. The patient underwent hemodialysis for 9.5 hours while receiving a modified “step 3” of protocolized N-acetylcysteine. Poison control advised using triple the usual N-acetylcysteine dose rate (normally 6.25 mg/kg/h for 16 hours) while off hemodialysis and 6-times the normal rate while on dialysis. This dose increase of N-acetylcysteine was arbitrarily chosen to our knowledge. Acetaminophen level decreased from a maximum measured level of 998 – 69 mg/L at hemodialysis discontinuation and 6 mg/L after 33 hours of modified “step 3” N-acetylcysteine. Concomitantly, the lactic acid level decreased from 9.1 to 2.0 mg/dL. The patient received 82.8 g of N-acetylcysteine total, or 0.99 g/kg in this case – triple the standard total dose. 

The patient was extubated within 24 hours with complete return to baseline functional capacity. Serum creatinine rose to baseline with resolved possible proximal tubular defect on repeat urinalysis while finishing N-acetylcysteine therapy post hemodialysis. His creatinine remained near baseline, and INR and liver enzymes normalized by hospital discharge the next day. Serum osmolarity was not measured. A timeline of events and laboratory measurements throughout the case are shown in Table 1. 

## Discussion 

This case demonstrates the therapeutic challenges, including interpreting clinical and laboratory findings, created by massive acetaminophen overdose and its ensuing treatment. Full-intensity hemodialysis was successfully used with markedly increased N-acetylcysteine dosing to rapidly reduce the acetaminophen level. Discriminating between markers of liver failure and alternative explanations for such findings are critical when weighing the risks and benefits of initiating various dialysis modalities when faced with toxin removal. In this case, no negative neurologic sequelae were observed, and development of subsequent hepatic injury was avoided. It is unknown to what degree each treatment, hemodialysis and the increased N-acetylcysteine dosing, contributed to the patient’s liver protection. Sivilotti et al. [14] reported the extraction ratio of N-acetylcysteine across a hemodialysis circuit above 70%. Therefore, removal of N-acetylcysteine by dialysis may be harmful unless N-acetylcysteine dosing is appropriately increased. The duration of hemodialysis in this case appears to be the longest reported in similar cases and was guided by the patient’s clinical improvement and substantial reduction in acetaminophen level [4]. 

It is unclear how the patient’s glomerular filtration rate was affected by these events due to apparent laboratory interference with creatinine measurement attributable to either acetaminophen or N-acetylcysteine, though occurring prior to modification of N-acetylcysteine dosing. To our knowledge, we are the first to highlight a laboratory interference such as this. While dialysis may be considered in cases of toxin exposure regardless of renal function, acute kidney injury is not uncommon in cases of acetaminophen overdose with or without acute liver failure [18]. Our most intriguing finding in this case, in addition to the apparent creatinine-lab interference, was the initial and quickly resolved polyuria with glucosuria and brief proteinuria. Neither the brief proteinuria nor the polyuria were consistent with typical tubular necrosis pathophysiology [3]. Rather, proteinuria appears better explained by acetaminophen-induced mitochondrial toxicity having caused transient tubular dysfunction [2, 3]. The observed polyuria may have had multiple causes: tubular dysfunction, hyperglycemia, and/or N-acetylcysteine-driven osmotic diuresis [19]. 

Alternatively, possible concurrent toxicity from “inactive” propylene glycol causing osmotic diuresis [20, 21] and lactic acidosis [22] cannot be ruled out. While listed by the U.S. Food and Drug Administration as “generally recognized as safe” [23], toxicity of non-intravenous medications containing propylene glycol have been reported [16, 24]. Management with hemodialysis would have been similar had such toxicity been confirmed in this case [25]. Therefore, it seems reasonable to suggest that in cases of very large overdoses, concomitant toxicity of “inactive” ingredients ought to be considered when interpreting findings in addition to co-ingestion of additional pills or substances. 

Despite anomalous findings and initial potential markers of liver injury, a combination of clinical suspicion and continued assessment allowed for the correct therapy to be utilized in this case. It did not appear that acute kidney injury occurred despite lacking accurate creatinine measurement from a rarely described lab interference, and hepatic or other end-organ damage was avoided with an overall excellent outcome for the patient despite a massive acetaminophen overdose. 

## Institutional review board 

This study was deemed not to be human subjects research by the University of Minnesota Institutional Review Board. 

## Funding 

None. 

## Conflict of interest 

None. 

**Table 1.** Timeline of events and laboratory measurements.

Event	Presents at outside ER	NAC started and intubation at outside ER	Labs repeated at outside ER	Transfer to our hospital	NAC dose increased and hemodialysis initiated	Labs repeated	Labs repeated and hemodialysis stopped	Labs repeated and patient extubated	Labs repeated and NAC stopped	Morning of discharge
Approximate time	Hour 1 (based on store receipt)	Unknown	Hour 5	Hour 7	Hour 10	Hours 13 – 17	Hour 19 (9.5 hours of dialysis)	Hour 21	Hours 37 – 43	Hour 64
Pertinent labs										
APAP	546		998	> 600	> 600	397, 205, 117	69	39	6	3
Lactate	4.2		9.2	9.1	6.0	4.4		2.0		
Na/K/ Cl/CO_2_/ AG/BUN/ Cr/Gl	135/4.2/ 103/18/ 14/13/ 0.95/150		137/4.4/ 113/< 10/ > 14/10/ 0.80/298	138/4.9/ 108/7/ 23/10/ < 0.14/327	142/4.3/ 112/10/ 20/7/ 0.14/221	142/3.4/ 108/19/ 15/2/ < 0.14/197		143/3.2/ 108/24/ 11/3/ 0.51/159	144/3.4/ 111/20/ 13/6/ 0.77/91	144/3.5/ 113/22/ 9/5/ 0.67/115
AST/ALT/ TB/AP	24/39/ 0.3/100			41/72/ 0.5/101	42/68/ 0.6/89	46/70/ 0.6/84		88/105/ 0.8/83	54/81/ 0.6/85	27/65/ 1.0/83
INR	1.0			1.41				1.45		1.14
NH_3_	55			49						
ABG: pH/ CO_2_/O_2_			6.99/ 42/161	7.05/ 28/168		7.43/ 32/166		7.49/ 34/102		
UA: sp gr/ pH/gluc/ alb/bld/ RBC/ WBC/LE/ nit/casts			1.025/ 5.0/100/ neg/neg/ N/A/ N/A/neg/ neg/N/A				1.025/ 8.5/150/ 10/neg/ 2/ 4/neg/ neg/hyaline		1.004/ 5.0/neg/ neg/small/ 7/ 1/neg/ neg/none	

NAC = N-acytelcysteine; APAP = acetaminophen level (µg/mL); lactate = lactic acid (mmol/L); Na = sodium (mmol/L); K = potassium (mmol/L); Cl = chloride (mmol/L); CO_2_ = bicarbonate (mmol/L); AG = anion gap (mmol/L); BUN = blood urea nitrogen (mg/dL); Cr = creatinine (mg/dL); Gl = glucose (mg/dL); AST = aspart transaminase (U/L); ALT = alanine transaminase (U/L); TB = total bilirubin (mg/dL); AP = alkaline phosphatase (U/L); INR = international normalized ratio; NH_3_ = ammonia (µmol/L); ABG = arterial blood gas (pH; CO_2_: PaCO_2_ (mmHg); O_2_: PaO_2_ (mmHg)); UA = urinalysis (sp gr = specific gravity; gluc = glucosuria; alb = albuminuria; bld = blood; RBC = red blood cells (per high-powered field); WBC = white blood cells (per high-powered field); LE = leukocyte esterase; nit = nitrite; casts). Neg = negative; pos = positive; N/A = not applicable as not asssessed.
